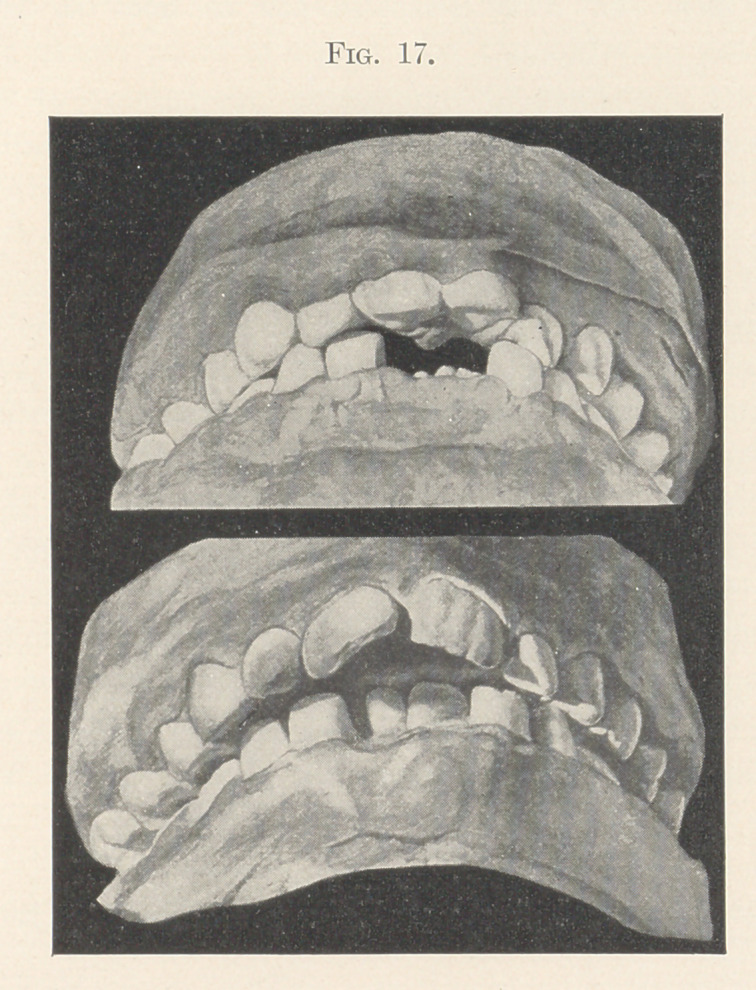# Medical Aspects of Dental Lesions

**Published:** 1903-08

**Authors:** Samuel A. Hopkins

**Affiliations:** Boston, Mass.


### MEDICAL ASPECTS OF DENTAL LESIONS.[1]

BY SAMUEL A. HOPKINS, M.D., D.D.S., BOSTON, MASS.

When we consider what an inviting field for medical investigation the mouth and the teeth furnish, it is difficult to understand why medical men have so long failed to appreciate the agency of the teeth in giving rise to and in aggravating diseases in other

---

[1] Read before The New York Institute of Stomatology, April 7, 1903.

FIG. 16.

FIG. 17.

organs. Not only have dental lesions been left for the dentist to treat, but the physician has too often failed to recognize their importance in diagnosis. Whether it be ears, throat, nose, eyes, glandular diseases, or diseases in more remote organs, almost every case reported as having its origin in diseased teeth bears with it a confession of failure on the part of the physician to make an early and successful diagnosis.

In the text-books we find brief allusions to the teeth as possible disturbers of neighboring organs, but except where the first dentition is made the scape-goat to bear the burden of every childish ailment, very little is said which would serve as a guide to the physician in making a diagnosis of diseases having their origin in the teeth and surrounding tissue. I can imagine no field which would yield such an abundant harvest if the reaping-knife of medical investigation could be thrust in.

There are no diseases of the ear, no diseases of the eye, no diseases of any other part of our anatomy, that can be exorcised so quickly as those which have their origin in diseases of the mouth and teeth.

Almost as soon as the diseased tooth has been discovered, behold! a magic wand is waved over the suffering patient. Pain gives way to amazement, amazement to gratitude, and gratitude to worship, and the intelligent physician who has been quick to diagnose and quick to act is the idol at whose shrine the grateful patient bends his knee.

It may, then, be well to refresh our memories by referring to some of the diseases which have their origin in dental disturbances, and, by way of reciprocity, to refer to some of those cases in which general diseases affect the teeth and associated parts. We may safely pass over the diseases which come with the eruption of the first teeth, because undue prominence is apt to be given to this perfectly normal physiological condition as a feature in the diseases of childhood. We are apt to forget that during this period not only is the mouth undergoing a change, but the follicular apparatus of the stomach and intestinal canal is undergoing rapid development, and a remarkable change is taking place in anticipation of the mixed diet which is to come with the development of the first teeth.

Not only the alimentary canal, but every organ in the body is undergoing rapid development, and the nervous system, particularly the cerebrospinal system, is in a condition of exceedingly high functional activity, wherein it responds quickly and in an exaggerated degree to an irritation that would go unnoticed at a less excitable period. It is no wonder that excess or irregularities in diet, be they ever so slight, should be able to throw the delicate mechanism of development out of gear. It is no wonder that any exposure to cold or contagion should find the system of the teething child an easy mark for the destructive arrows of disease. It is no wonder if at this period the least departure from hygienic principles should be punished with quickly following illness, nor is it any wonder that some irritation should be manifested as a result of the efforts of the teeth to force their way through the gums. It is well to remember, however, that the diseases which we are apt to attribute to first dentition would seldom arise were it not for the fact that the condition of the infant organism is so susceptible at this period that the slightest influence has exaggerated power to disturb the entire system.

It is not quite the same with second dentition, for here, with the exception of those mysterious changes which take place at puberty, changes which are psychological as well as physiological, and which we can neither fully understand nor explain,—with this exception we have the body unaffected by unusual changes in the progress of its development. With the eruption of the sixth-year molars we frequently have a period of general depression and nervous irritation. If we recognize this condition, it will serve to make us more patient and forbearing if the little victim gives way to his feelings and is not quite as amenable to discipline as we could wish. Usually this passes away as soon as the teeth come through, and treatment is seldom required. It does, however, happen at this period that because the gum is swollen and painful to chew upon the child will bolt his food, washing it down with water or milk, or will refuse altogether to eat solid food, thus giving rise to serious digestive disturbances. In a like manner, when the bicuspids come through, pushing before them the molars of the deciduous set, digestive disturbances again may arise from the inability or unwillingness of the little patient to chew his food. It will be a great gain to the child if the physician is quick to discover the source of these slight troubles and take the necessary steps to relieve them. Acting in the same way, a carious and painful temporary tooth will break up the habit of mastication and bring on diseases of the stomach and intestines that are capable of wrecking the happiness of a lifetime.

It is imperative, then, that we should be on the lookout for disorders of second dentition, and, moreover, it is exceedingly important that the temporary teeth should be kept free from disease in order that mastication may be properly performed. The physician will find, also, that many cases of stomatitis of an ulcerative type may be accounted for by the eruption of sixth-year molars and bicuspid teeth. These cases all yield to simple treatment if the source of the irritation has once been discovered.

Ordinarily the eruption of the twelfth-year molars is unnoticed, yet it would be well to remember that they are capable of setting up a severe train of nervous and constitutional symptoms. This is particularly true in cases where the jaw is short and the teeth are crowded. If hysteria, fretfulness, loss of appetite, irritation of the eyes and ears, with anæmia, occur about the tenth to the twelfth year, it will pay to have a careful examination made to discover if a lower second molar painfully pushing its way towards the surface may not give rise to these symptoms. Frequently the X-ray will have to be employed to give the exact position of the tooth in the jaw and its relation to the other teeth, as upon this knowledge will depend the treatment. So much for the disorders which the eruption of the teeth give rise to.

Before taking up the somewhat formidable list of evils which the teeth are capable of producing, it may be well to mention the fact that not only do diseases of the teeth produce constitutional disturbances, but constitutional disturbances may reveal themselves in severely painful attacks of odontalgia. We have all, I fancy, a certain chamber of horrors in our memory, the door of which we keep closed even to our intimate friends. Occasionally we are obliged to go in ourselves and view the unlovely skeletons of our earlier mistakes. It is not a pleasant duty, and we close the door as quickly as possible and pray that the repulsive adornments may not be increased. Most of us who have practised dentistry remember having been baffled almost to the point of desperation by severe pain, often recurrent and sometimes long continued, in a tooth or teeth that were apparently sound. Often mistaking such a condition for the pain arising from exostosis or from the deposit of pulp-stones, we have destroyed the pulp, only to have the pain continue or pass into an adjoining tooth. Too late we have discovered that malaria or rheumatism, or some other general disease, had started up a train of symptoms that culminated in this painful condition of the teeth.

It will never be easy to make the diagnosis, but it will often serve us in time of need to remember that such reflexes may occur not only from malaria and rheumatism, but from diseases of the eye and stomach as well. During the menstrual period the teeth are frequently sensitive and uncomfortable. Toothache during the nausea of sea-sickness is of common occurrence, and is relieved by the vomiting which follows. Hysteria finds the teeth a favorite part of the body in which to end its erratic course, and a disorder of the brain may readily involve a perfectly sound set of teeth. As the human organism is a very perfect unit, it is difficult to imagine any serious affection of one part that may not give rise to reflex symptoms in another, and while in the present state of our knowledge it is impossible to classify these symptoms with accuracy, yet a proper realization of the broad principle of unity in all bodily ailments may help us to avoid serious mistakes in diagnosis.

We now come to a consideration of diseases of neighboring organs induced by carious teeth, or by abscesses or other abnormal conditions of the teeth and surrounding tissue.

One of the most offensive conditions which diseases of the teeth give rise to is that which is accompanied by chronic discharge from the nose. Such a discharge may be due to syphilis, lupus, necroses of the bones or cartilages, or inflammation of the frontal and maxillary sinuses, but is very commonly found to have its origin in a diseased tooth. A glance at the relation of the upper incisor teeth to the nasal fossa and the relation of the posterior teeth to the antrum of Highmore will immediately discover the means by which an alveolar abscess, or even less violent inflammation of the pericemental membrane, may be communicated to the frontal and maxillary sinuses and other neighboring organs and produce a train of symptoms difficult to distinguish from chronic nasal catarrh.

So frequently is a diseased tooth the seat of the trouble that it should be a matter of routine to have an examination of the teeth made in every such case. I do not mean a cursory glance by the physician, but the most searching tests of all the upper teeth should be made by a competent dentist. After excluding syphilis and lupus, the chances of the teeth being involved are very great. In the case of the front teeth the discharge is usually not very offensive. When the antrum is involved by diseased conditions of the posterior teeth the thickening of the mucous membrane blocks up the opening so that the discharge is retained until it becomes in some instances as offensive as that which accompanies syphilis. There is a long train of general symptoms, chill, fever, prostration, etc., which I need not go into, but I wish to emphasize the following suggestion, and beg of you to accept it as a rule of practice.

Given chronic nasal catarrh, always have the teeth carefully examined. Another suggestion in this connection,—having removed the cause, do not do too much in the way of treatment. There is a dangerous tendency to keep up the irritation by the too frequent use of the probe and syringe. Secure the drainage and, after one or two washings with non-irritating antiseptics, give nature a chance. It is true that she may be more interested in raising a group of pyogenic bacteria than in the cure of the patient, but there is a good prospect of her effecting a cure if not interfered with too much.

Besides the nasal inflammation which arises from a diseased anterior upper tooth, inflammation extending from one of the lower teeth, particularly one of the lower wisdom-teeth, has also been known to give rise to inflammation of the nasal mucous membrane and a consequent discharge from the nose. We may also have an inflammation from a tooth, which inflammation will pass over the roof of the mouth and palatal arch, and, extending to the pharynx, cause catarrhal pharyngitis, or it may pass to the middle fossæ of the nose, causing hyperæmia of the turbinated bodies and acute rhinitis.

Consideration of the subject of nasal catarrh brings us to the subject of otitis media. I do not know but the aural specialist will consider my statement too sweeping, but I am led to believe from my own observation and from a study of the literature of the subject that disease of the middle ear is almost always preceded by a catarrhal disease of the nasal mucous membrane, and this, we have just seen, is frequently provoked by a diseased tooth. Therefore, otitis media may be, and frequently is, caused by a diseased tooth. Disease of the ear may be reflex in its nature or may be the direct extension of inflammation from a tooth. In some cases this inflammation may result in the closing of the Eustachian tube. The closing of the tube produces a partial vacuum in the canal and tympanic cavity. The pressure of the outside air against the membrana tympani drives it inward and stretches it to its utmost capacity. This tension not only causes severe pain, but there is naturally pressure on the blood-vessels. Venous engorgement occurs, and is followed by congestion, inflammation, suppuration, and rupture of the drum-head.

While many cases of direct continuation of the inflammation from the tooth to the ear may be cited, the number caused by reflex action through the vasomotor centres is far greater than we have ever imagined. We know that the normal caliber and tone of the arteries is maintained by the action of the vasomotor centre. The sympathetic ganglia have the power of receiving impressions from one direction and reflexly referring them to an entirely different organ. The sympathetic ganglia are closely connected with the general vasomotor centre, and physiologists have shown that the vascularity of a part may be augmented or inhibited, first, by irritation or stimulation applied to the part itself; secondly, by stimulation of some other part acting through the general vasomotor centre; or, thirdly, by stimulation acting directly on the vasomotor centre. If you keep in mind the fact that the middle coat of the arteries is largely made up of circular muscular fibres, and remember that nerve-fibres belonging to the sympathetic system are distributed to these blood-vessels, you will comprehend how the blood-supply may be altered, not only by the reflex action of an irritation at some remote part, but even by a wave of emotion or a passing thought as exemplified in the action of blushing. The familiar physiological experiment of dividing the cervical sympathetic in a rabbit relaxes the blood-vessels of the ear where the changes can be beautifully observed. The arteries become engorged with blood and minute arteries that had escaped attention become easily distinguished. If the cut nerve be stimulated, the blood disappears and the ear becomes even paler than normal.

We have only to remember that inflammation is the result of congestion and venous engorgement, and we can see plainly how an irritation from an exposed pulp, from pulp-stones, from an abscess, from an impacted wisdom-tooth, from pyorrhœa alveolaris, or other diseased conditions of the gum and mucous membrane of the mouth may be transmitted by the nerve coming from the tooth through some of the sympathetic ganglia to the nerves supplying the blood-vessels, not only of the middle ear, but to the external auditory meatus as well, and it will readily be seen that there is scarcely a diseased condition of the ear that may not be produced by dental irritation. Twenty years ago Dr. Samuel Sexton, of New York, published, in the *American Journal of Medical Sciences,* what is to my mind the best essay ever written on "The Affections of the Ear arising from Diseases of the Teeth." In reviewing the records of fifteen hundred cases of aural disease he says, "Perhaps one-third owe their origin or continuance, in a greater or less degree, to diseases of the teeth."

It had been my intention to complete this paper by referring to diseases of the eye, the stomach, and the nervous system, which frequently arise from diseases of the teeth, and are cured by the proper treatment of those organs. I have said enough, however, to make it plain that such an association must exist, and to warn the practitioner to be ever on the alert to trace to their proper origin the many unexplained and elusive disturbances now too often overlooked.

I am obliged to close this part of my paper in a somewhat unfinished state, in order to give you an account of some bacteriological work which I feel will be of interest to the dental profession, because it brings out some important characteristics of growth which certain mouth bacteria possess, and has an intimate connection with the prevention of dental and medical lesions.

In recording the experiments which follow, the writer wishes to acknowledge his indebtedness to Dr. Harold C. Ernst, Professor of Bacteriology in the Harvard Medical School, who gave the writer all the facilities of his laboratory and made many very valuable suggestions during the progress of the work.

It has long been known that nearly every common form of bacteria, both of the pathogenic and non-pathogenic variety, finds its way at some time or other into the human mouth. Many forms which appear in cultures or cover-slip preparations from the mouth must be looked upon as but temporary—perhaps only momentary—lodgers in the oral cavity, while others can be observed with relative frequency. Still others are so generally and constantly to be found in the mouth that while it would hardly be safe to say that they were actually indigenous, it can be asserted without hesitation that there they find conditions suitable to their growth and rapid development. Leaving out of consideration the non-pathogenic varieties and several still unclassified organisms that are slightly pathogenic for small animals, there remain several varieties having undoubted power to produce disease that occur with sufficient frequency in the human mouth to arrest our attention and suggest a possible danger.

The staphylococcus pyogenes aureus is perhaps the most common of the pyogenic forms. Black found this organism in seventy per cent. of the mouths he examined. The observations of the writer would lead him to look upon this estimate as much too high, but the organism is found with sufficient frequency to entitle it to be classified with those bacteria commonly found in the human mouth. The micrococcus tetragenus is found in the sputum of tuberculous patients in nearly every case (Koch, *Mittheilungen an das kaiserliche Gesundheitsamt*, Bd. xi. S. 42), but whether it plays a part of any importance in connection with that disease has not as yet been satisfactorily determined. It is found also in perfectly healthy mouths with varying frequency, and it has been stated that saliva containing this bacillus is fatal to mice and guinea-pigs. In those cases studied by the writer this did not always prove to be the case, but Biondi and others have noticed this fatal action. This action is not to be confounded with the fatal action of the micrococcus of sputum septicæmia or micrococcus lanceolatus, as it is variously called. While the latter organism is, according to Fränkel and Weichselbaum, almost always present in the mouths of those suffering from croupous pneumonia, it is by no means uncommon in the mouths of healthy persons. This organism, the micrococcus lanceolatus, was discovered by Sternberg in 1880, who found it in the oral cavity of about twenty per cent. of the healthy mouths examined. The fact that it has been found in the pleura, in the middle ear, in the frontal sinus, and in the antrum suggests many possibilities of evil. In the experiments about to be described it was exceedingly baffling in its variation in pathogenic power.

Taking these three pathogenic mouth forms, because of their wide distribution and the ease with which they could be found, experiments were begun to explain if possible the facts noted by many observers, that these and other pathogenic bacteria varied greatly in their virulence in different mouths and also in the same mouth at different periods. It was believed that by studying the pathogenic properties of the mouth forms under varying conditions light might be thrown upon the questions of variations in the severity of disease which are so often observed. It was hoped also to discover some explanation to account for the difference in the virulence of bacteria from different mouths and from the same mouths at different times.

This, it was hoped, might lead to the discovery of some inhibitive force which retarded bacterial activity and which would lead to the prevention of disease. Experiments were naturally first directed towards the saliva, with the hope of finding that some inhibitive action existed in the secretions that would account for the variation in the action of the bacteria of the mouth. This, however, did not prove to be the case. Unsterilized saliva from a healthy mouth did seem to restrict the action of the aureus and the micrococcus lanceolatus by causing increased phagocytosis in the animals experimented upon. Sterilized saliva, however, had no such action, although great care was taken to sterilize it by long exposure to a temperature not high enough to affect the ptyalin. This temperature was found to be slightly below 65° C. Saliva sterilized in this way or by means of a Chamberlain filter (both methods being exceedingly laborious and requiring great care and patience) was not found to have any effect upon the pathogenic action of the bacteria referred to, either when injected in connection with the bacteria or when injected separately, either previous to or after the inoculation of the animal; nor was the growth of these forms perceptibly altered or their virulence changed by the addition of sterilized saliva to the culture medium in which they were growing. It was evident that while the saliva might in an unsterilized condition contain many innocent forms of bacteria which would awaken the phagocytes to action or give rise to enzymes which might change the action of pathogenic forms, it was probable that the saliva freed from bacteria had no such property. It is only fair to state that this result does not accord with the experiments of Sanarelli. (*Centralblatt für Bakteriologie,* Bd. x., 1891, p. 817.)

After much time and labor had been expended in this somewhat fruitless investigation of the possible inhibitory action of saliva, attention was directed to the culture material which, in the form of food particles, desquamated epithelium, etc., exists almost constantly in the mouth and which by alteration in its character or by any increase or diminution in its amount might serve to inhibit or to increase the growth of the three pathogenic forms used in these experiments.

It had been observed by other investigators that not only the rapidity of growth but the pathogenic properties of all bacteria depend greatly upon the amount and kind of the culture medium used. That this was true of these mouth forms under consideration was easily determined so far as it applied to growths on artificial media, and the author was encouraged to believe that the same would prove true when they were studied in their natural condition in the human mouth; that is to say, that a form would be more numerous and the virulence would be greater under conditions which favored its growth in the mouth, and that it would become less active and less numerous when deprived of nutrition.

In order to find cases containing the three organisms experimented with, a great many patients both in private practice and in the dispensary had to be examined, and many hundred cultures and cover-glass preparations had to be made, and the author is greatly indebted to Dr. John Coolidge, then assistant in Bacteriology in the Harvard Medical School, for examining and classifying many of the cultures of mouth bacteria which were used in this work. Work was begun with the staphylococcus pyogenes aureus, and although it anticipates the results somewhat, it may be stated that when once found in the mouth this form was more persistent than either of the other two examined. In Case No. 1, staphylococcus pyogenes aureus was found in the mouth in great abundance. Several cavities containing pus were discovered in the gum margin around the necks of the teeth, and these pockets contained many aureus forms. Masses were also found adhering to the teeth. Cultures made at this time showed this pus-producing form to be extremely virulent.

It is well known that the subcutaneous inoculation of this bacterium in lower animals does not always produce a suppurative process, and large quantities of a bouillon culture may be introduced into the abdominal cavity without producing inflammation, unless something which acts as a direct irritant be introduced at the same time. When, however, the organism is injected directly into the circulation the results leave no doubt as to its action. For this reason rabbits were used in the present experiment. In one animal 0.2 cubic centimetre of a bouillon culture was introduced into the venous circulation of the rabbit's ear, and in another rabbit inoculated at the same time the same amount of a watery suspension of the organism was used. In the first animal death followed in a little less than three days, and the second animal died about twelve hours later.

The appearance displayed at the autopsy of these animals was in every way typical. The pericardial sac was distended by a gelatinous substance, and yellow minute abscesses were seen in the myocardium. The diaphragm and kidneys were studded with these yellow spots. The muscles also showed great numbers of these spots. The liver and brain apparently were not affected. Cultures and cover slip preparations from these minute abscesses left no doubt as to the cause of death. Staphylococcus pyogenes aureus was found in pure cultures. Treatment was now directed to the patient in whose mouth this organism had been found, and for three weeks the most rigid cleanliness was enforced. The pus pockets were syringed out daily with pyrozone, and twice applications of nitrate of silver were made. Under this treatment a marked improvement was made, although an absolute cure was by no means accomplished. The patient was enjoined to cleanse the teeth after every meal and to remove the food particles as quickly and as thoroughly as possible. This precaution was insisted upon because it seemed reasonable to believe that food particles remaining in the mouth would furnish an excellent medium for the development of bacteria.

At the end of three weeks cultures were taken from as nearly as possible the same spot in the mouth as that from which the previous culture had been taken. This was upon the buccal surface of the upper left second molar about an eighth of an inch from the opening of one of the pus-pockets referred to. It is interesting to note that at this time almost all forms of bacteria in this mouth as shown by numerous cover slips were far less numerous than when examinations were made three weeks before. The diminution was particularly marked in the thread-like forms and in the spirilla, but just what significance may be attached to this observation the writer is at present unable to say. The cultures of the aureus made at this time were treated exactly as those taken three weeks before, and showed but slight variations in their development upon artificial media except in one particular. The chromogenic action of the first cultures was much more marked, and the characteristic color appeared slightly earlier than in the later cultures. It is, however, not to be inferred that chromogenic action is any indication of virulence.

Two rabbits of approximately the same weight as those used in the earlier experiments were selected and inoculated as before, care being taken that in every particular the operations should be a repetition of those performed three weeks earlier, the only difference being that in these latter experiments the cultures were taken, as has been said, after the mouth had been scrupulously cleaned and treated for three weeks.

The animal inoculated with a watery suspension of the organism died at the end of seven days, while the animal inoculated with a bouillon culture survived. In the case of the surviving animal the only symptoms noticed, except a slight dulness, was an increase in the amount of urine passed. The autopsy upon the dead animal showed, in addition to the yellow abscesses referred to in the previous case, a somewhat marked peritonitis.

This experiment was repeated with bacteria taken from two other mouths, with but slight variation in the result. In Case No. 2 only one animal died of the two inoculated with cultures from the uncared-for mouth, while both animals inoculated after the mouth had been cared for for a month survived and apparently experienced no great discomfort.

In Case No. 3 both animals died on the fourth day when inoculated with cultures from the unclean mouth, and cultures taken after three weeks' care killed one animal in five days, and the other died on the ninth. This case is of greater significance than would appear from a simple statement of results, because, owing to the illness of her child and the poverty of her surroundings, it was almost impossible for this patient to greatly improve the condition of her mouth, and there was little difference in its condition between the first and second inoculations.

Other experiments were made by finding the organism (as is sometimes possible) in a clean, well-cared-for mouth and comparing its virulence with that of the same organism taken from a filthy, uncared-for mouth in which pus-pockets and abscesses abound. These experiments, which will cover a large number of cases, are not yet completed, but sufficient evidence has been accumulated to make clear the fact that the staphylococcus pyogenes aureus is more virulent when taken from filthy mouths than when taken from mouths that receive constant care.

Experiments with the micrococcus tetragenus were much simplified by the fact that guinea-pigs and white mice are quite susceptible and could therefore be used for inoculation. It is interesting to note that gray mice are not susceptible to this bacterium. The organism, as has been said, is almost always found in tuberculous patients and is frequently seen in healthy mouths.

Throughout these experiments care was taken not to confound this organism with the micrococcus tetragenus subflavus which is sometimes found in nasal mucus and which may find its way into the mouth. While the micrococcus tetragenus grows but slowly on nutrient gelatin, the micrococcus subflavus does not grow at all on that medium. Other marked differences make it impossible to confound the two except by gross carelessness.

It has been said that guinea-pigs and white mice were susceptible to the micrococcus tetragenus, but in the case of white mice death was often delayed until the eighth or ninth day, while in the case of guinea-pigs a local abscess was often the only result of the inoculation. At other times death occurred from general infection. When this occurred, whether in guinea-pigs or white mice, there were few characteristic signs in any of the organs examined at the autopsy. Microscopic examinations of the blood, however, revealed the presence of the organism, and the inoculation of other susceptible animals with a drop of blood or a bit of tissue from the dead animal would reproduce the disease in the animal inoculated.

It was discovered that while the organism under consideration was easily found in the mouths of tuberculous patients, it was by no means as common in well-cared-for mouths as we had been led to suppose. One of the chief difficulties in experimenting with this form was that while it was to be found in fully ten per cent. of healthy mouths that did not receive special care,—such mouths, for instance, as are met with in dispensary practice,—yet in private practice, among people of cleanly habits, who carefully brushed the teeth, the organism was by no means common. The writer failed to find it in more than two per cent. of the latter cases. Another difficulty encountered was the fact that when discovered in the mouth of one of these dispensary patients, if the patient could be induced to go to the dental dispensary and have the mouth thoroughly cleaned and put in order, and if he could then be persuaded to wash and cleanse his mouth several times a day for a fortnight, the organism would disappear except (as was to be supposed) in the mouth of the tuberculous patient, where its number greatly diminished under careful cleansing of the mouth. The aureus was much more persistent and resisted careful cleansing of the mouth for many weeks; indeed, in some cases it seemed nearly impossible to get rid of it, so tenaciously did it adhere to the teeth and gums.

The following cases will serve to illustrate the variations in virulence of the micrococcus tetragenus under different conditions.

Case No. 1 was a tuberculous patient with teeth in good order and a mouth clean and well cared for. Cultures taken from this mouth and introduced into white mice caused death in from three to five days. Of two guinea-pigs inoculated with the same culture, one died in five days, while the other survived, but showed local abscess. The organism was recovered in these as in the following cases.

Case No. 2 was also a tuberculous patient, but, unlike the first case, the mouth was shockingly neglected and contained several badly diseased teeth, while several were missing. Cultures were made as in the previous case, and two white mice and two guinea-pigs were used for inoculation purposes. As a result one white mouse died in two days and one in four days, while one guinea-pig died in five days and the other survived until the ninth day, when he died from a mixed infection. The micrococcus seemed slightly more virulent in this case than in Case No. 1, but the difference was hardly great enough to be significant.

This mouth was thoroughly cleaned, the abscessed teeth extracted, and the patient instructed to cleanse the mouth thoroughly several times a day. This she did faithfully for two weeks, and cultures were again taken and inoculations made as before. There was no perceptible diminution in the virulence of the cultures, the animals dying in about the same time as when the culture was taken from an unclean mouth. Cover-glass preparations showed a great reduction in number of nearly all forms of bacteria after the mouth had been carefully cleaned for a period of two weeks. It was regretted that this case could not be watched for a longer period, but the patient was ordered to seek another climate, and the case was lost sight of.

Case No. 3 was a patient in good health, with a clean, well-cared-for mouth. There were few coccus forms present, although a number of rod forms, both straight and curved, could be seen in cover-glass preparations. The culture was obtained early in the morning before the mouth was cleansed. Many previous attempts to obtain cultures of the micrococcus tetragenus from this mouth had failed. Following inoculation, one white mouse died in six days and one in seven. Both guinea-pigs survived. One had an abscess at point of inoculation, but the organism could not be recovered from this animal. Inoculations were made from the same culture five days after the first inoculations were made. In this second series only one white mouse succumbed and that at the end of the seventh day. The virulence of the organism grows less the longer it is grown on artificial media. It increases by being passed through susceptible animals.

Case No. 4 was from the mouth of a patient who was suffering from several abscessed teeth, and whose mouth was in a totally uncared-for condition; otherwise, the patient was in excellent health. Many coccus forms were present in the mouth. The tetragenous form was isolated and the usual inoculations were made, with the result that one white mouse died in four days and one in five days. Both guinea-pigs died on the seventh day.

Case No. 5, the patient who figured in Case No. 4, was induced to carefully cleanse his mouth, paying especial attention to his tongue and teeth. His teeth were properly treated and put in fair order. Eighteen days after this treatment had begun, and the same number of days after the first culture was taken for inoculation (Case No. 4), the attempt was made to find the organism. Eleven cultures were made from different parts of the mouth, and the bacterium was found in two only. One of these was taken from the crypt of the tonsils and the other was taken from the gum surrounding one of the dead teeth that was still undergoing treatment. Inoculation with the culture taken from the tonsils caused the death of one white mouse on the seventh day. The second mouse escaped from the cage on the fourth day and could not be recovered. One guinea-pig died on the fifth day from a mixed infection, while the other survived. Inoculation of mice and guinea-pigs from the culture taken from the neighborhood of the diseased tooth was not fatal in any case.

Many other cases were studied, and these would perhaps be of interest to the student of bacteriology, but enough has been said to point to the following conclusions: That the micrococcus tetragenus is more active when taken from the mouth of a tuberculous patient than from the mouth of a healthy person. That cleansing the mouth in tuberculous patients greatly lessens the number, while it does not always lessen the virulence of this bacterium. It is possible that a longer period of cleanliness might give a more favorable result.

In mouths of healthy individuals this organism occurs with greater frequency and in greater numbers, and is much more virulent when the mouth is uncared for than when it is habitually well cared for.

The organism will usually disappear from a mouth in a few weeks if the mouth is properly cleansed several times a day during that period. This does not apply to tuberculous cases, although, as has been said, a reduction in the number is usually effected by cleansing.

The micrococcus lanceolatus is variously described under many names, as, Diplococcus pneumoniæ (Weichselbaum); Streptococcus lanceolatus pasteuri (Gameleia); Bacillus salivarius septicus (Biondi); Micrococcus pneumoniæ crouposæ (Fränkel); etc. It is found in the saliva in many diseased conditions as its variety of names would indicate, but it also occurs in the saliva of healthy individuals, and this saliva is often fatal to small animals.

Sternberg called attention to this bacterium, which he discovered in the blood of rabbits which had been previously inoculated with saliva from his own mouth. At about the same time (1880), Pasteur found it in the saliva of a child suffering with hydrophobia.

A number of other writers have made numerous experiments with this organism, and their results may be summed up by saying that it is not constant in the mouth, but appears and disappears as if by accident, and that saliva containing this microbe varies in virulence under different conditions.

It was necessary to make but a few experiments to demonstrate a fact which had been already pointed out by Sternberg and others, that micrococcus lanceolatus loses its pathogenic property to a marked degree when it is grown on artificial media. It is also more easily destroyed by antiseptics than most bacteria, and its growth is retarded and its virulence lessened by antiseptics which are not powerful enough to completely destroy the organism.

Its pathogenic properties are quickly revived by passing it through susceptible animals. Emmerich, in 1891, demonstrated the immunizing action of this bacterium, and in the writer's experiments it was found that an animal once inoculated with this organism without fatal result was thereafter immune to very large doses. How long the immunity lasts has not been ascertained.

It was to be expected, then, that mouth cleanliness would cause a disappearance of this microbe in many cases, and this the writer found to be true. It is probably the easiest of all pathogenic mouth forms to get rid of. Absolute cleanliness of the mouth, including tongue and teeth, with the frequent use of an antiseptic mouth-wash would, in a majority of instances, cause the entire disappearance of this bacterium in from three to fourteen days.

The same treatment, when it did not actually destroy the microbe, would render the saliva which contained it *non-pathogenic,* provided that the saliva itself was normal. A further physiological and chemical study of saliva secreted under various conditions of health will probably throw light on the question of its action in encouraging or retarding the growth of bacteria. In the present experiments the writer was unable to enter into the study of that phase of the question, but it was observed in a general way that saliva which was clear and watery offered less encouragement to the development of bacteria than that which was thick and viscid and which apparently contained large quantities of mucus and broken-down epithelial cells.

If it be true, as can now be scarcely doubted, that this microbe is the excitant of croupous pneumonia, it is undoubtedly true also that the infection is derived from the mouth in a vast majority of cases. We have, then, a most important factor in preventive medicine in mouth cleanliness, and it can be asserted with a degree of positiveness that is fully borne out by experiments and by clinical experience that this disease might be almost eliminated from human ills were it possible to keep the mouth in a clean, healthy condition.

This, we know, is impossible. People can not be persuaded, except possibly when the disease is exceedingly prevalent and the danger from exposure is imminent, to spend the time necessary to guard against the likelihood of mouth infection, but there is another aspect of the question which will certainly appeal to the physician anxious to prevent disease.

It is well known that croupous pneumonia frequently follows other diseases. It is one of the dreaded sequelæ of measles, whooping-cough, and typhoid fever, and appears to develop sometimes from a severe cold. This, we know, is not strictly true, for it is not possible for one disease to turn into another, since the characteristic pathogenic properties of a given bacterium do not depart greatly from well-defined lines. It is, however, true that a system disordered by what we term a cold, or having its resisting power lowered by one disease, is peculiarly susceptible to the attacks of another; and, as in the case of croupous pneumonia, if the microbe of the disease is lurking in the mouth it finds the system peculiarly susceptible to its attacks after one of the aforementioned diseases has lowered the vitality of the patient.

Many physicians now recognize in a degree the importance of cleansing the mouth, and the educated nurse, if she be a careful woman, will brush regularly her patient's teeth, because she has found that this simple act greatly adds to his comfort. Yet it is true that comparatively few physicians or nurses have learned to look upon cleanliness of the mouth as an important factor in preventing complicating diseases.

If, however, the experiments presented have not been wrongly interpreted, the pneumonia germ is one of the easiest to destroy, and mouth cleanliness will go far to reduce the disease to a minimum.

If diphtheria and other pathogenic forms which find their way into the mouth are influenced by the conditions which have been shown to affect the virulence of the bacteria experimented upon, then thorough mouth cleanliness will be found to be our greatest safeguard against disease.

## Figures and Tables

**Fig. 16. f1:**
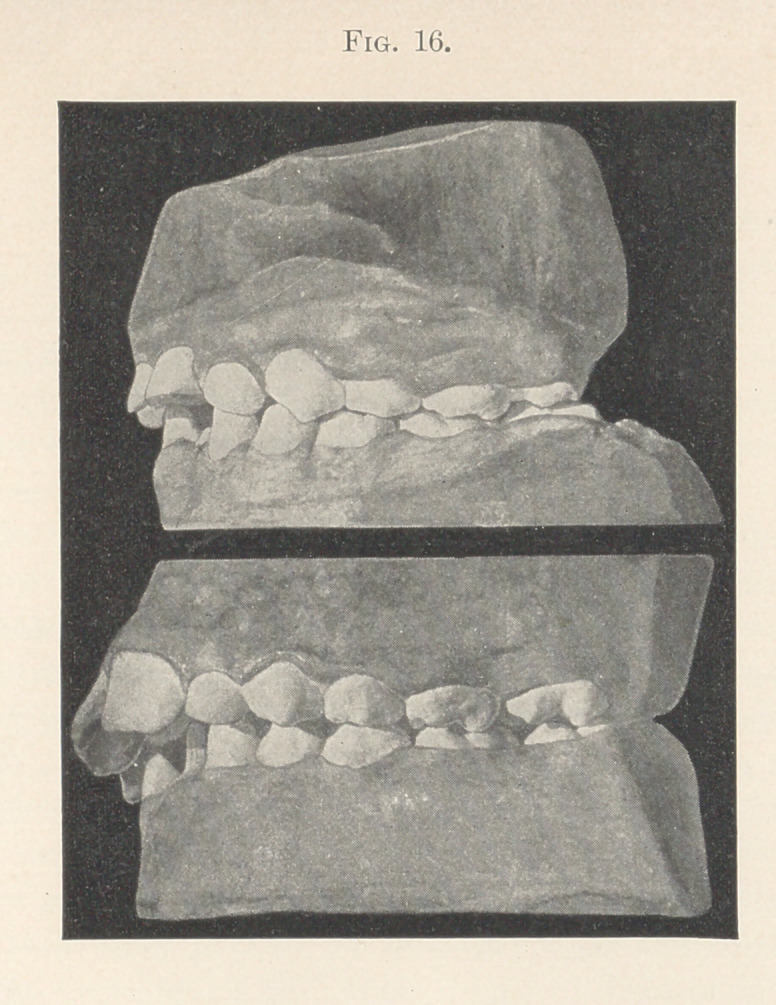


**Fig. 17. f2:**